# eCPR Combined With Therapeutic Hypothermia Could Improve Survival and Neurologic Outcomes for Patients With Cardiac Arrest: A Meta-Analysis

**DOI:** 10.3389/fcvm.2021.703567

**Published:** 2021-08-13

**Authors:** Jingwei Duan, Qingbian Ma, Changju Zhu, Yuanchao Shi, Baomin Duan

**Affiliations:** ^1^Emergency Department, Peking University Third Hospital, Beijing, China; ^2^Emergency Department, The First Affiliated Hospital of Zhengzhou University, Zhengzhou, China; ^3^The First Clinical Medicine School, Lanzhou University, Lanzhou, China; ^4^Emergency Department, Kaifeng Centre Hospital, Kaifeng, China

**Keywords:** extracorporeal cardiopulmonary resuscitation, therapeutic hypothermia, survival, neurologic outcomes, meta-analysis

## Abstract

**Background:** Extracorporeal membrane oxygenation with CPR (eCPR) or therapeutic hypothermia (TH) seems to be a very effective CPR strategy to save patients with cardiac arrest (CA). Furthermore, the subsequent post-CA neurologic outcomes have become the focus. Therefore, there is an urgent need to find a way to improve survival and neurologic outcomes for CA.

**Objective:** We conducted this meta-analysis to find a more suitable CPR strategy for patients with CA.

**Method:** We searched four online databases (PubMed, Embase, CENTRAL, and Web of Science). From an initial 1,436 articles, 23 studies were eligible into this meta-analysis, including a total of 2,035 patients.

**Results:** eCPR combined with TH significantly improved the short-term (at discharge or 28 days) survival [OR = 2.27, 95% CIs (1.60–3.23), *p* < 0.00001] and neurologic outcomes [OR = 2.60, 95% CIs (1.92–3.52), *p* < 0.00001). At 3 months of follow-up, the results of survival [OR = 3.36, 95% CIs (1.65–6.85), *p* < 0.0008] and favorable neurologic outcomes [OR = 3.02, 95% CIs (1.38–6.63), *p* < 0.006] were the same as above. Furthermore, there was no difference in any bleeding needed intervention [OR = 1.33, 95% CIs (0.09–1.96), *p* = 0.16] between two groups.

**Conclusions:** From this meta-analysis, we found that eCPR combined with TH might be a more suitable CPR strategy for patients with CA in improving survival and neurologic outcomes, and eCPR with TH did not increase the risk of bleeding. Furthermore, single-arm meta-analyses showed a plausible way of temperature and occasion of TH.

## Introduction

With the popularization of cardiopulmonary resuscitation (CPR), an increasing number of patients could survive with cardiac arrest (CA). However, the mortality and morbidity of CA still continued to increase, and the inflection point has not yet been reached (1). Despite patients could survive after successful resuscitation, the bad neurologic outcome has constituted another great obstacle, which brought huge burden for family and society (2). The probability of good neurologic outcome from CA decreases with every minute of CPR, especially in patients with refractory cardiopulmonary arrest who did not achieve return of spontaneous circulation (ROSC) (3). Accordingly, it is of great necessity to find an optimal strategy of CPR, which cannot only improve survival but also neurologic outcome.

Extracorporeal membrane oxygenation (ECMO), as a circulatory and pulmonary support device, was used to replace the function of the heart and lungs. CPR under the ECMO support was first proposed in 1966 and reached superior outcome of survival compared with conventional cardiac pulmonary resuscitation (cCPR) (4). The American Heart Association (AHA) 2020 guidelines of CPR recommended eCPR for patients after CA, if condition permits (5). While extracorporeal cardiopulmonary resuscitation (eCPR) could support sufficient perfusion for end organs such as the kidneys, liver, and brain, it seemed not to improve the neurologic outcome for patients after CA (6).

During CA, the neurocyte is in an extreme anoxic condition. Because the neurocyte could not acquire adenosine triphosphate *via* anaerobic glycolysis, a few minutes of anoxia could cause irreversible necrosis of the neurocyte. First, from an animal model, scientists found that mild hypothermia (temperature 34°C) could significantly mitigate brain damage by reducing cerebral oxygen consumption and metabolism. Furthermore, after successful resuscitation, combining with therapeutic hypothermia (TH) could indeed improve neurologic outcome (7, 8). In the contemporary society, the standard of successful resuscitation for patients with CA seems to be both survival and good neurologic outcome. Therefore, we conduct this meta-analysis, which is aimed to find a more suitable strategy of resuscitation.

## Materials and Methods

We performed this meta-analysis according to the Preferred Reporting Items for Systematic Reviews and Meta-Analyses (PRISMA) statement. The PICO (patient, intervention, control, and outcome) strategy was used in this analysis. For the patient who had CA, the intervention was eCPR with TH; the control received eCPR without TH, and the outcomes were survival and neurologic outcome at discharge or 28 days. Based on this strategy, we schemed the search strategy.

### Search Strategy

We searched four online databases (PubMed, Embase, CENTRAL, and Web of Science) from January 1, 2000 to December 31, 2020. We also searched the references of relevant studies, reviews, editorials, and letters.

Search term keywords included, “Extracorporeal Membrane Oxygenation or Extracorporeal Membrane Oxygenations or Membrane Oxygenation, Extracorporeal or Oxygenation, Extracorporeal Membrane or ECMO Treatment or ECMO Treatments or Treatment, ECMO or ECLS Treatment or ECLS Treatments or Treatment, ECLS or ECMO Extracorporeal Membrane Oxygenation or Extracorporeal Life Support or Extracorporeal Life Supports or Life Support, Extracorporeal or Venoarterial ECMO or ECMO, Venoarterial or Venoarterial ECMOs or Venoarterial Extracorporeal or Membrane Oxygenation,” “Cardiopulmonary resuscitation or Resuscitation, Cardiopulmonary or CPR or Cardio-Pulmonary Resuscitation or Cardio Pulmonary Resuscitation or Resuscitation, Cardio-Pulmonary or Code Blue or Mouth-to-Mouth Resuscitation or Mouth to Mouth Resuscitation or Mouth-to-Mouth Resuscitations or Resuscitation, Mouth-to-Mouth or Resuscitations, Mouth-to-Mouth or Basic Cardiac Life Support or Life Support, Basic Cardiac,” “Heart Arrest or Arrest, Heart or Cardiac Arrest or Arrest, Cardiac or Asystole or Asystoles or Cardiopulmonary Arrest or Arrest, Cardiopulmonary,” and “Hypothermia, Induced or Therapeutic Hypothermia or Hypothermia, Therapeutic or Targeted Temperature Management or Targeted Temperature Managements or Induced Hypothermia or Moderate Hypothermia, Induced or Induced Moderate Hypothermia or Induced Moderate Hypothermias or Moderate Hypothermias, Induced or Mild Hypothermia, Induced or Induced Mild Hypothermia or Induced Mild Hypothermias or Mild Hypothermias, Induced or Hypothermia.” After searching, all articles were imported into Endnote X9 to manage citations and screen for duplicate citations.

### Strategy of Study Selection

The inclusion criteria were as follows: (a) All adult patients received eCPR after CA. (b) At least one group of patients received TH (the target temperature should be controlled from 32 to 36°C) regardless of before or after ROSC. (c) The study should aim to compare the survival or neurologic outcome of eCPR with TH or not. (d) The outcomes of survival or neurologic outcome could be extracted (e) if study did not aim to compare eCPR with TH or not. It, at least, consisted of a subgroup that could be extracted to compare the survival or neurologic outcome of eCPR with TH or not.

The exclusion criteria were as follows: (a) The population of the study was children, neonates, or pupillage, (b) any animal study, (c) single-arm study without control group. (d) Although the design of the study is in accordance with the inclusion criteria, it had insufficient data. (e) full article was not found; (f) all case series or case report.

### Endpoint Design

The primary endpoints were defined as survival and neurologic outcome at discharge or 28 days (short term). The neurologic outcome was estimated by the Cerebral Performance Category (CPC). The CPC 1–2 was defined as good neurologic outcome, and the CPC 3–5 was defined as bad neurologic outcome (9).

The secondary endpoints were defined as survival and neurologic outcome at 3 months. The criteria of neurologic outcome are the same as above. Furthermore, we also conducted eight single-arm meta-analyses to compare the short-term survival and neurologic outcome of different temperatures (32°C ≤ temperature ≤ 34°C; 34°C < temperature ≤ 36°C) and different occasions of TH (before or after ROSC). Furthermore, any bleeding needed medical intervention was compared between eCPR with TH and eCPR without TH.

### Data Extraction

**Figure 1:**
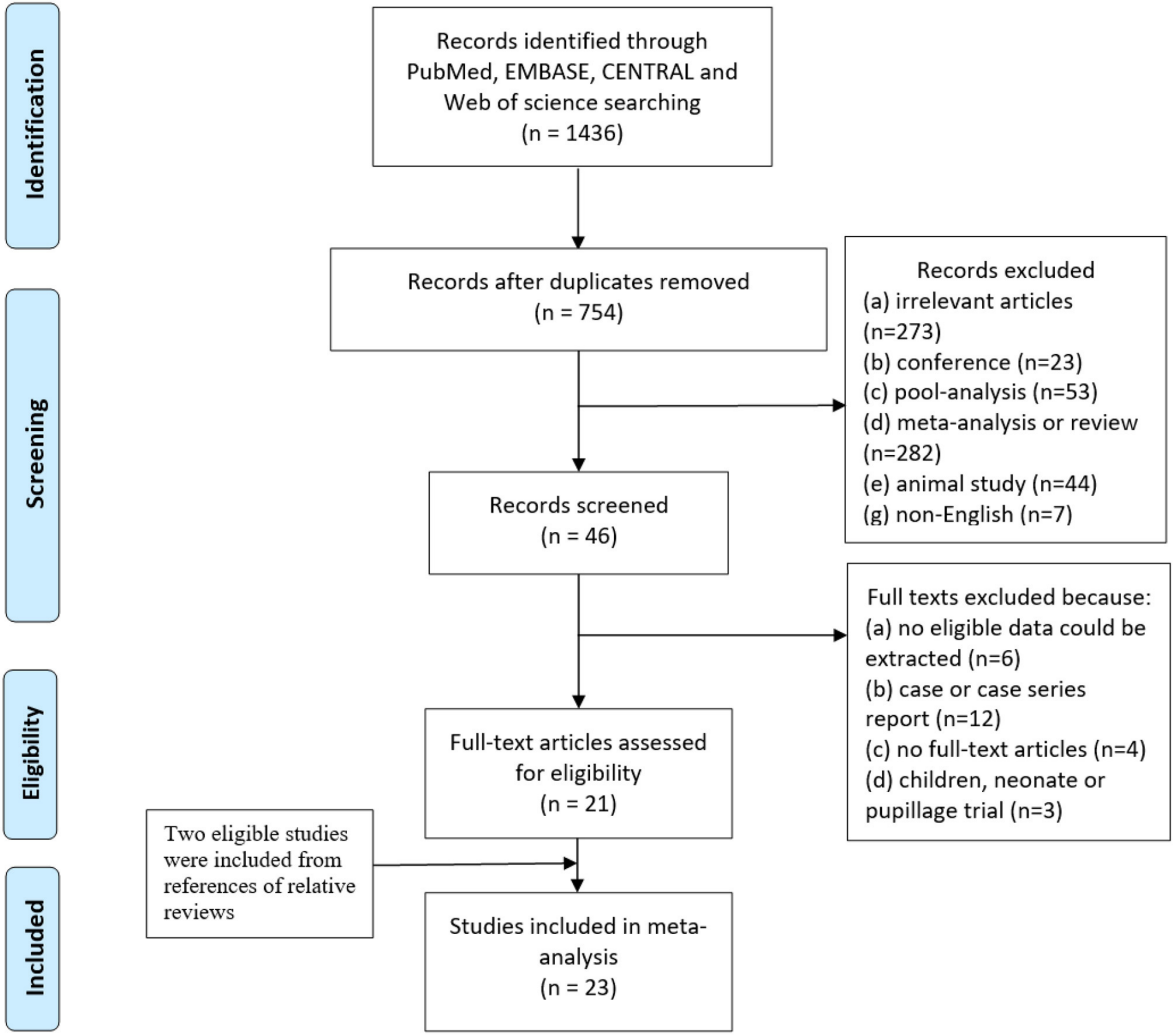
Flow diagram according to preferred reporting items for systematic reviews and meta-analyses (PRISMA) statement.

Initially, two trained investigators independently screened the titles and abstracts of all the articles. If there was a disagreement, a third person was consulted to settle the disagreement. After preliminary screening, the full texts of the remaining publications were read. Finally, all the included trials were confirmed as eligible by trying to contact the corresponding authors. The details are shown in Figure 1.

### Quality Assessment of Eligible Studies

The Cochrane Handbook (version 5.1.0) was used to assess the quality of randomized controlled trials (RCTs) and risk of bias. The Newcastle–Ottawa Scale (NOS) was used to assess the quality of retrospective trials. The study score >7 was regarded as high quality (10). The bias and quality of the included studies are shown in Supplementary Tables 1, 2 and Supplementary Figure 1.

### Statistical Analysis

We used the *I*^2^ statistic to assess heterogeneity; values <25, 25–50, and >50% were defined as low, moderate, and high heterogeneity, respectively. A fixed-effects model was used to obtain odds ratio (OR) and 95% confidence intervals (CIs). If the *I*^2^ > 25%, the random effects model was used. Sensitivity analysis was performed by calculating the number of patients in each trial as a percentage of the total number of patients to determine the weight of each trial in the overall results of the meta-analysis. The *p*-value was used to determine whether there was a significant difference between the two groups. No significant difference was considered when *p* > 0.05. When *p* ≤ 0.05, a significant difference was considered. In addition, there was a low-to-moderate significant difference when 0.05 ≥ *p* > 0.001 and a highly significant difference when *p* ≤ 0.001. The OR was also calculated to judge whether the intervention measure decreased the risk of adverse events. In the forest plot, when we set adverse events as comparable endpoints, the right side indicated protective factors with RR > 1, the left side indicated adverse factors with RR <1, and the center line indicated no effect, with RR = 1. The publication bias was analyzed and represented by a funnel plot (Supplementary Figures 11–15).

Additional eight single-arm meta-analyses were also conducted in RevMan 5.3. Based on Chen et al., the result from RevMan should be recalculated [Pf = OR/(1 + OR); LL = LLOR/(1 + LLOR); UL = ULOR/(1 + ULOR)] (11). The above analysis was conducted in Review Manage (RevMan 5.3).

## Results

### Study Selection

The search strategy initially identified 1.436 studies from four online databases. Duplicate studies (682) were removed, and 754 of the remaining 682 studies were excluded after reading the titles and abstracts because they did not meet the inclusion criteria or met the exclusion criteria. A total of 46 studies were screened by reading the full text, and 24 studies were excluded. Additionally, two studies were included in this meta-analysis by references of relative reviews. Twenty-four studies were eligible for inclusion, but when we extracted the data from these studies, we found that no valid data could be extracted from pre-specified subgroup in one study. Finally, 23 studies were included in this meta-analysis (Figure 1).

### Characteristics of the Included Studies

The pooled data from eligible studies included 2,035 patients, with 917 patients in the eCPR with the TH group and 1,118 patients in the eCPR without the TH group. However, most of the studies were conducted in Korea and Japan. The proportion of survival and favorable neurologic outcomes for patients receiving eCPR with TH ranged from 19.0–86.2% to 7.9–46.7%, respectively. All patients of the 12 studies were from OHCA and one study involved all patients with shockable rhythm and acute coronary syndromes that caused CA. Since not all studies reported our pre-specified primary endpoints, only 20 studies were included in the analysis of short-term survival with 747 patients in the eCPR with the TH group and 919 patients in the eCPR without the TH group, and 21 were included in the analysis of the short-term neurologic outcomes with 848 patients in the eCPR with the TH group and 962 patients in the eCPR without the TH group. The detailed characteristics of these studies are presented in Tables 1, 2.

**Table 1:** Demographic characteristics of eligible studies.

**References**	**Region**	**Male**	**Age**	**OHCA**	**Witnessed arrest**	**Shockable rhythm**	**Bystander CPR**	**Tc temperature control**	**TH case**	**Arrest to eCPR time (min)**	**ACS cause CA**	**Follow-up (day)**
Choi et al. (12)	Korea	7 (70.0)	57	10 (100)	10 (100)	3 (30.0)	8 (80.0)	33°C	6 (60.0)	49	n/a	30
Dennis et al. (13)	Australia	27 (73.0)	54	12 (32.4)	27 (73.0)	19 (51.4)	30 (81.1)	34°C	22 (59.5)	45	11 (29.7)	30
Fjølner et al. (14)	Denmark	12 (57.1)	56	21 (100)	6 (28.6)	9 (42.9)	4 (19.0)	36°C	9 (42.9)	54	10 (47.6)	Discharge
Goto et al. (15)	America	122 (84.7)	63	144 (100)	25 (17.4)	88 (61.1)	54 (37.5)	34°C	63 (43.8)	54	100 (69.4)	30
Han et al. (16)	Korea	74 (74.0)	55	75 (75.0)	86 (86.0)	54 (56.0)	73 (73.0)	35°C	26 (26.0)	74	n/a	30
Jouffroy et al. (17)	France	30 (65.2)	52	46 (100)	46 (100)	n/a	46 (100)	32–34°C	29 (63.0)	88	27 (58.7)	28
Kagawa et al. (18)	Japan	55 (71.4)	62	39 (50.6)	67 (87.0)	29 (37.7)	63 (81.8)	33°C	25 (32.5)	42	43 (55.8)	365
Kagawa et al. (19)	Japan	70 (81.4)	63	44 (52.2)	77 (89.5)	46 (53.5)	67 (77.9)	35°C	32 (37.2)	49	86 (100)	365
Kagawa et al. (20)	Japan	64 (73.6)	62	54 (62.1)	82 (94.3)	40 (46.0)	66 (75.9)	34°C	48 (55.2)	n/a	48 (55.2)	90
Kim et al. (21)	Korea	40 (76.9)	54	52 (100)	42 (80.8)	31 (59.6)	22 (42.3)	33°C	14 (26.9)	34	44 (84.6)	90
Kim et al. (22)	Korea	69 (68.3)	55	22 (21.8)	n/a	45 (44.6)	98 (97.0)	33–34°C	25 (24.8)	45	84 (83.2)	Discharge
Lee et al. (23)	Korea	20 (87.0)	55	23 (100)	n/a	10 (43.5)	14 (60.9)	33–34°C	18 (70.3)	84	15 (65.2)	30
Maekawa et al. (24)	Japan	44 (83.0)	54	53 (100)	53 (100)	32 (60.4)	29 (54.7)	34°C	26 (49.1)	49	21 (39.6)	90
Mecklenburg et al. (25)	Germany	46 (69.7)	51	n/a	n/a	n/a	n/a	32–34°C	36 (54.5)	185	27 (40.9)	28
Nagao et al. (26)	Japan	148 (86.5)	59	171 (100)	171 (100)	143 (83.6)	94 (55.0)	34°C	45 (26.3)	56	131 (76.6)	30
Nagao et al. (26)	Japan	119 (78.8)	60	151 (100)	151 (100)	151 (100)	n/a	34°C	138 (91.4)	62	151 (100)	30
Otani et al. (27)	Japan	115 (85.2)	65	135 (100)	135 (100)	87 (64.4)	74 (54.8)	34°C	28 (20.7)	47	64 (47.4)	Discharge
Pang et al. (28)	Singapore	17 (81.0)	53	3 (14.3)	9 (42.9)	7 (33.3)	n/a	34°C	9 (42.9)	46	17 (82.0)	180
Pang et al. (29)	Singapore	62 (78.5)	50	7 (7.6)	73 (92.4)	33 (41.8)	n/a	34°C	14 (17.7)	32	62 (78.5)	60
Ryu et al. (30)	Korea	174 (63.5)	62	24 (8.8)	272 (99.3)	79 (28.8)	262 (95.6)	34°C	36 (13.1)	36	104 (38.0)	Discharge
Sakamoto et al. (31)	Japan	235 (90.4)	56	260 (100)	186 (71.5)	260 (100)	127 (48.8)	32–34°C	231 (88.8)	60	165 (63.6)	180
Schober et al. (32)	Austria	5 (71.4)	46	7 (100)	6 (85.7)	4 (57.1)	2 (28.6)	35°C	3 (42.9)	93	2 (28.6)	Discharge
Yukawa et al. (33)	Japan	65 (82.3)	59	79 (100)	21 (26.6)	58 (73.4)	46 (58.2)	34°C	50 (63.3)	45	26 (32.9)	Discharge

**Table 2:** Basic characteristics of eligible studies.

**References**	**Design of study**	**eCPR + TH (*n*)**	**eCPR alone (*n*)**	**Pre-eCPR pH**	**Pre-eCPR lactate (mmol/L)**	**Survival outcomes**	**Neurologic outcomes**	**Bleeding outcomes**
Choi et al. (12)	Retrospective	6	4	n/a	n/a	Yes	Yes	No
Dennis et al. (13)	Retrospective	15	22	7.17	9.4	Yes	Yes	Yes
Fjølner et al. (14)	Retrospective	9	12	6.45	17.2	Yes	Yes	No
Goto et al. (15)	Retrospective cohort	63	71	6.95	12.5	Yes	Yes	No
Han et al. (16)	Retrospective cohort	26	74	n/a	12.5	Yes	Yes	Yes
Jouffroy et al. (17)	Prospective cohort	29	17	n/a	n/a	Yes	No	No
Kagawa et al. (18)	Retrospective	21	18	7.13	n/a	Yes	Yes	Yes
Kagawa et al. (19)	Retrospective	32	54	n/a	n/a	Yes	No	No
Kagawa et al. (20)	Retrospective	48	39	n/a	n/a	Yes	Yes	Yes
Kim et al. (21)	Retrospective	14	38	6.98	11.6	Yes	Yes	Yes
Kim et al. (22)	Retrospective	25	76	7.03	6.5	Yes	Yes	Yes
Lee et al. (23)	Retrospective	18	5	7.01	9.7	Yes	No	Yes
Maekawa et al. (24)	Retrospective	26	26	7.00	17.2	Yes	Yes	Yes
Mecklenburg et al. (25)	Retrospective	36	30	n/a	7.6	Yes	No	Yes
Nagao et al. (26)	Prospective cohort	86	85	n/a	n/a	Yes	Yes	No
Nagao et al. (26)	Prospective cohort	138	13	n/a	n/a	No	Yes	No
Otani et al. (27)	Retrospective	28	107	6.90	13.2	No	Yes	No
Pang et al. (28)	RCT	9	12	n/a	n/a	Yes	Yes	Yes
Pang et al. (29)	Retrospective	14	65	n/a	n/a	Yes	Yes	Yes
Ryu et al. (30)	Retrospective	36	238	n/a	7.9	Yes	Yes	No
Sakamoto et al. (31)	Prospective observational	231	29	n/a	n/a	Yes	Yes	No
Schober et al. (32)	Retrospective	3	4	6.95	12.0	Yes	Yes	No
Yukawa et al. (33)	Retrospective	50	29	n/a	14.0	No	Yes	No

### Primary Endpoints

**Figure 2:**
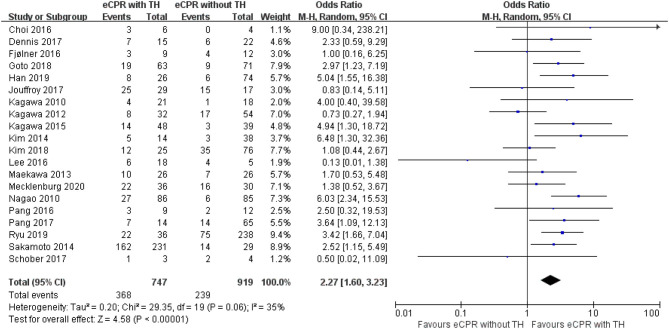
Forest plot for survival at discharge or 28 days (eCPR, extracorporeal cardiopulmonary resuscitation; TH, therapeutic hypothermia).

**Figure 3:**
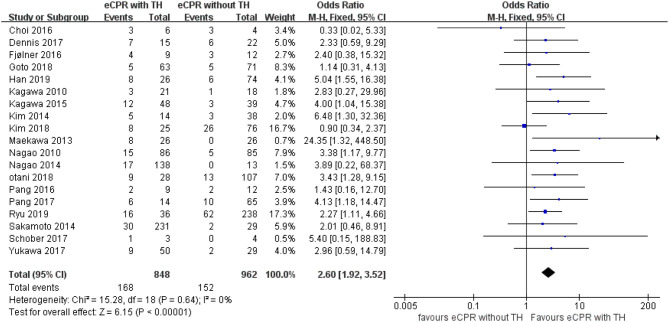
Forest plot for favorable neurologic outcomes at discharge or 28 days (eCPR, extracorporeal cardiopulmonary resuscitation; TH, therapeutic hypothermia).

A random-effects model was used to compare short-term survival. eCPR combined with TH significantly improved short-term survival for patients after CA than eCPR alone [OR = 2.27, 95% CIs (1.60–3.23), *p* < 0.00001; *I*^2^ = 35%). Regarding short-term neurologic outcomes, compared with eCPR alone, eCPR with TH significantly improved the neurologic outcomes [OR = 2.60, 95% CIs (1.92–3.52), *p* < 0.00001; *I*^2^ = 0% fixed-effects model] (Figures 2, 3).

### Secondary Endpoints

**Figure 4:**
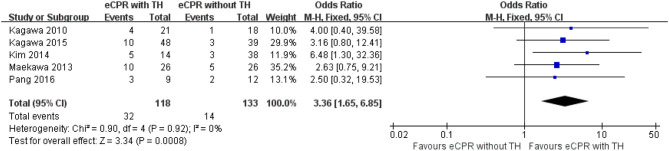
Forest plot for survival at 3 months (eCPR, extracorporeal cardiopulmonary resuscitation; TH, therapeutic hypothermia).

**Figure 5:**
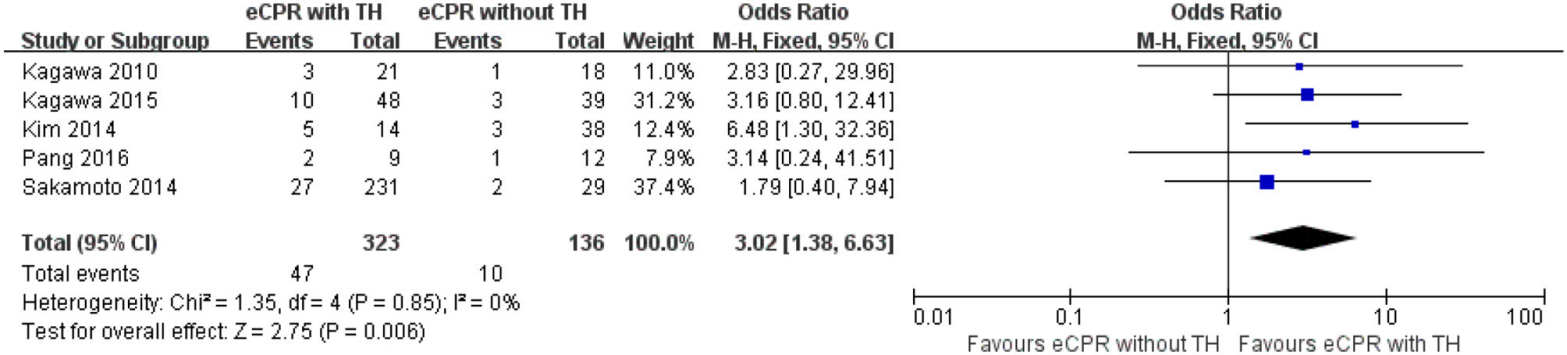
Forest plot for favorable neurologic outcomes at 3 months (eCPR, extracorporeal cardiopulmonary resuscitation; TH, therapeutic hypothermia).

Regarding 3-month survival, five studies were included, with 118 patients in the eCPR with the TH group and 133 patients in the eCPR without the TH group. eCPR combined with TH was superior to eCPR alone for patients after CA [OR = 3.36, 95% CIs (1.65–6.85), *p* < 0.0008; *I*^2^ = 0% fixed-effects model]. Five other studies were included to compare the 3-month neurologic outcomes, with 323 patients in the eCPR with the TH group and 136 patients in the eCPR without the TH group. eCPR with TH was still superior to eCPR alone [OR = 3.02, 95% CIs (1.38–6.63), *p* < 0.006; *I*^2^ = 0% fixed-effects model] (Figures 4, 5).

**Figure 6:**
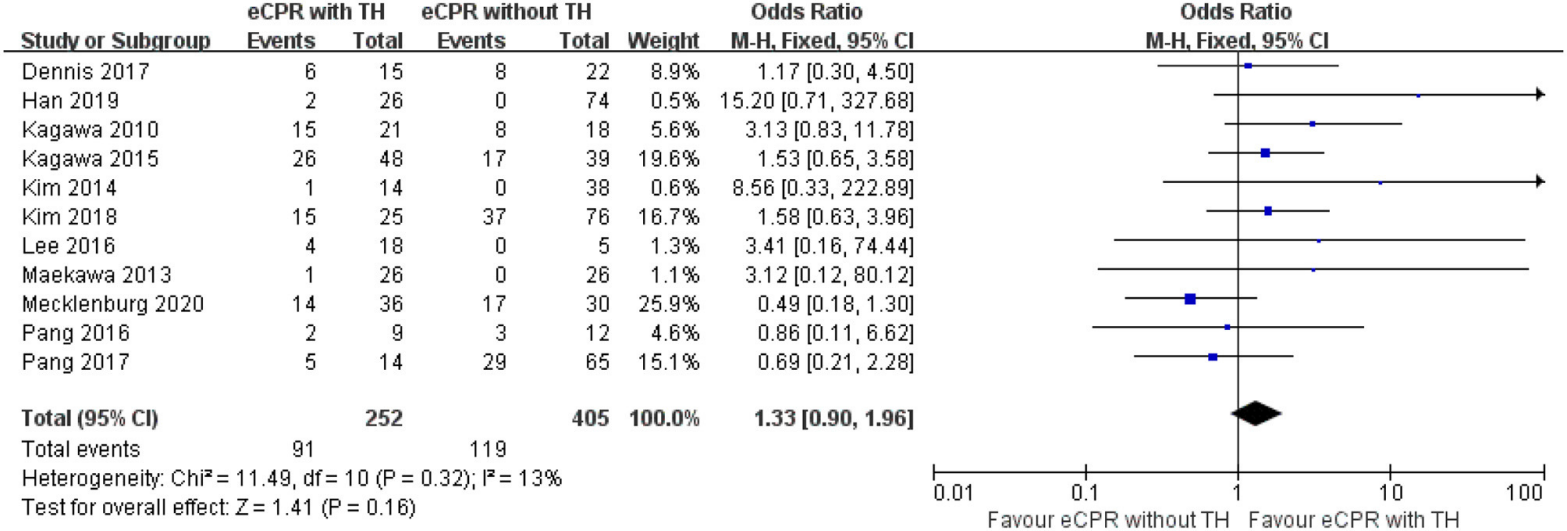
Forest plot for bleeding risk between eCPR alone and eCPR + TH (eCPR, extracorporeal cardiopulmonary resuscitation; TH, therapeutic hypothermia).

Regarding complication of any bleeding needed medical intervention, 11 studies were included, with 252 patients in the eCPR with the TH group and 405 patients in the eCPR without the TH group. eCPR with TH did not increase this risk [OR = 1.33, 95% CIs (0.09–1.96), *p* = 0.16; *I*^2^ = 13% fixed-effects model) (Figure 6).

### Results of Single-Arm Meta-Analyses

Eight single-arm meta-analyses were also conducted to preliminarily compare survival and neurologic outcomes of different temperature and occasion of TH. Regarding survival, the temperature of TH <34°C and performing TH before return of spontaneous circulation (ROSC) was superior to the temperature of TH over 34°C and performing TH after ROSC (30 vs. 24%; 35 vs. 27%, respectively). Regarding neurologic outcomes, performing TH after ROSC was superior to before ROSC (27 vs. 23%). However, temperature differences did not seem to affect neurologic outcomes (24 vs. 24%) (Table 3, Supplementary Figures 4–9).

**Table 3:** After adjusting the results of the single-arm meta-analyses.

**Name**	**OR_**adj.**_**	**95% CI_**adj.**_**
Survival of temperature >34°C	0.24	−0.92–0.52
Survival of temperature ≤ 34°C	0.30	0.22–0.35
Good neurologic outcome of temperature >34°C	0.24	−0.92–0.52
Good neurologic outcome of temperature ≤ 34°C	0.24	0.17–0.30
survival of TH before ROSC	0.35	0.17–0.47
survival of TH after ROSC	0.27	0.23–0.31
good neurologic outcome of TH before ROSC	0.23	−0.39–0.47
good neurologic outcome of TH after ROSC	0.27	0.17–0.35

### Sensitivity Analysis

The heterogeneity of short-term survival was high, and the *I*^2^-value was 35%. After reanalysis of eligible studies, we found that survival rate found by Lee et al. (23) was high in eCPR without TH (4/5 80%). Furthermore, it was found by Kagawa et al. (19) that ACS caused CA in all patients. For these patients, time of onset to open the culprit coronary artery was the most significant (34). After removing these two studies, the heterogeneity of short-term survival was very small, and the *I*^2^-value was only 2%. Moreover, eCPR with TH still significantly improved the short-term survival than eCPR without TH [OR = 2.68, 95% CIs (2.05–3.51), *p* < 0.00001; fixed-effects model] (Supplementary Figure 10).

## Discussion

We conducted this meta-analysis to primarily evaluate the short-term survival and neurologic outcomes of patients after CA receiving eCPR with TH or not. We found that eCPR combined with TH could significantly improve the survival and neurologic outcomes than eCPR alone for patients after CA. Furthermore, combining with TH did not increase the risk of any bleeding needed medical intervention. Therefore, eCPR combined with TH might be a more suitable CPR strategy for patients after CA in improving survival and neurologic outcomes.

When clinicians first found that ECMO assisting CPR could more effectively save lives for patients after CA, eCPR has been widely used in the clinic (4). Compared with cCPR, several studies have demonstrated that eCPR indeed improved survival for patients after CA (6, 35, 36). This advantage of eCPR might attribute to it that it can break this vicious cycle by inducing the circulation of perfectly oxygenated blood (22). Moreover, under ECMO assistance, external chest compression became more effective. However, there were still a large number of patients losing their life (26, 37). We still faced many challenges for the treatment of CA. The most significant challenge is prognosis of neurologic function. The worse neurologic outcome or even becoming a vegetable is not only a burden for society but also for family (38). For patients who survive from cardiac arrest, one of the most important ways to maintain favorable neurologic outcomes is to significantly reduce the metabolism of neurocyte as much as possible in a short time. Since a neurocyte could not acquire energy from an anoxic environment, the metabolite of a neurocyte would result in irreversible injury. Accordingly, arterial blood lactate level as a reflection of the body's metabolic level has a strong correlation with the prognosis. When scientists realized that low temperature could prolong the preservation of food, they kept trying to use the same method to preserve cells. Previous studies showed that TH (temperature from 32 to 36°C) could effectively save neurologic function by decreasing cerebral metabolism. Moreover, under anoxic environment, TH also saves the function of other organs, such as the heart, kidney, liver, and so on. Thus, TH seems to not only improve neurologic outcomes but also survival. This guess has been shown in several previous studies. However, a recent study has argued that normothermia had similar survival and neurologic outcomes to that of TH (39) because the body temperature is lower than normal after CA, although we also regarded the lower body temperature as a self-protection mechanism. Therefore, eCPR combined with TH might be a suitable CPR strategy for patients after CA. Recently, a registered trial showed that, compared with eCPR alone, eCPR with TH significantly improves the survival and neurologic outcome (50.0 vs. 21.5%, *p* = 0.029; 42.9 vs. 15.4%, *p* = 0.020, respectively). Furthermore, arterial lactate level as a reflection of the body's metabolic level has a strong correlation with the prognosis (40). eCPR combined with TH also significantly decreased the arterial lactate level than eCPR alone [4 mmol/L (3–7) vs. 8 (5–12), *p* < 0.01] (25).

Despite eCPR being a more effective CPR strategy, complications are frequent, and severe bleeding episodes occur in up to 40% of patients on eCPR (41). At the same time, anticoagulation is essential during eCPR to counteract possible thromboembolism. Moreover, coagulation may be disturbed due to post-cardiac arrest syndrome (42). This complication might be aggravated when combing with TH because a lower temperature could impact the coagulation system, especially between 32 and 34°C. Previous studies showed that a lower temperature could decrease the activity of clotting enzymes and concentration of fibrinogen and inhibit the function of platelets. Thus, clinicians are concerned about bleeding after eCPR with TH. Recently, the Mecklenburg et al. study showed that eCPR with TH did not increase the risk of major bleeding (25). However, a previous study demonstrated that a target temperature <34° significantly increases the risk of any bleeding needed medical intervention than the target temperature >34°C (53 vs. 31%, *p* < 0.001). In our meta-analysis, although we did not compare the risk of bleeding under different temperatures of TH, the overall risk of any bleeding needed medical intervention was not increased in ePCR with TH. Accordingly, further studies aiming to confirm and identify an optimal anticoagulant treatment for patients receiving eCPR with TH are also needed. Regarding other complications of eCPR with TH, such as sepsis, limb ischemia, and other device-related complications, they do not seem to be specific post-eCPR (43).

Both eCPR and TH are beneficial for patients after CA. Nevertheless, another problem is the temperature and occasion of TH. Previous studies just declared that hypothermia could improve survival and neurologic outcomes, but did not declare that benefits would be better for CA if the body temperature was in a more precise range of 32 to 34°C, 35 to 36°C, or other. Moreover, which is the best time for intervention of TH, after ROSC or before ROSC? The latest guideline did not explain these problems (5). A previous study showed that therapeutically cooling arrest patients to 33°C significantly improved survival (69 vs. 31%, *p* = 0.02) but did not improve neurologic outcomes (40 vs. 32%, *p* = 0.09) than cooling to 35°C (13). However, there is no study that compares with TH before or after ROSC in improving survival and neurologic outcomes. Despite we only conducted eight single-arm meta-analysis, the results from them seem to preliminarily explain these problems. We found that keeping the body temperature below 34°C could improve survival, but it did not benefit for neurologic outcomes. Furthermore, using TH before ROSC might improve the success of resuscitation, but this strategy did not improve neurologic outcomes. The above results provided a reasonable evidence for further studies to find an optimal temperature and occasion of TH for CA. Finally, we did not neglect a significant problem, and no randomized controlled trial was included in this meta-analysis. A biggest concern of retrospective and cohort studies is that clinicians intended to choose more invasive interventions (eCPR with TH) for patients who they think are more likely to survive (44). Since the results of retrospective and cohort studies are the only source of evidence, we do not completely deny the results from them.

## Limitations

This meta-analysis had the following limitations: Most of the eligible studies were retrospective and cohort studies. Therefore, there might be some bias in patient allocation in the retrospective studies. Furthermore, 12 studies only included OHCA patients, and 2 studies only included patients with shockable rhythm and ACS caused CA. Because the etiology is single, it might cause bias of prognosis. A total of 16 studies were from Asia. Therefore, the results might be biased by ethnic homogeneity and regional differences in medical standards. Most of the data were extracted from a subgroup of eligible studies, and the sample from some studies was too small. Thus, bias due to the above reasons might be inevitable. Finally, since baseline characteristics of these studies were partially missing, the meta-regression could not be conducted.

## Conclusion

Based on this meta-analysis, we drew the following conclusions: eCPR combined with TH might improve short-term survival and neurologic outcomes than eCPR alone for patients after CA. After extended follow-up of up to 3 months, patients receiving eCPR with TH still have favorable survival and neurologic outcomes. Furthermore, compared with eCPR alone, eCPR with TH did not increase the risk of any needed medical intervention bleeding. From single-arm meta-analyses, the temperature and occasion of TH seem to be related to survival and neurologic outcomes for patients after CA. It would provide an evidence for further studies. Of course, further studies aiming to confirm and identify an optimal CPR strategy for patients after CA are also needed.

## Data Availability Statement

The raw data supporting the conclusions of this article will be made available by the authors, without undue reservation.

## Author Contributions

QM: conceptualization, methodology, and supervision. JD: data curation and writing—original draft preparation. CZ: writing—reviewing and editing. YS: visualization and collection. BD: software and validation. All authors contributed to the article and approved the submitted version.

## Conflict of Interest

The authors declare that the research was conducted in the absence of any commercial or financial relationships that could be construed as a potential conflict of interest.

## Publisher's Note

All claims expressed in this article are solely those of the authors and do not necessarily represent those of their affiliated organizations, or those of the publisher, the editors and the reviewers. Any product that may be evaluated in this article, or claim that may be made by its manufacturer, is not guaranteed or endorsed by the publisher.
