# Supplementary material for: eCPR Combined With Therapeutic Hypothermia Could Improve Survival and Neurologic Outcomes for Patients With Cardiac Arrest: A Meta-Analysis
Source: Front Cardiovasc Med. 2021 Aug 13;8:703567. doi: 10.3389/fcvm.2021.703567 (PMC8414549; doi:10.3389/fcvm.2021.703567)
Supplement: Supplementary file 1 [file Data_Sheet_1.docx]

**Supplementary File**

| Study name | Selection | Comparability | exposure | NOS score |
| --- | --- | --- | --- | --- |
| **Choi 2016** | 3 | 2 | 3 | 8 |
| **Dennis 2016** | 2 | 2 | 3 | 7 |
| **Fjølner 2016** | 2 | 2 | 3 | 7 |
| **Kagawa 2010** | 3 | 2 | 2 | 7 |
| **Kagawa 2012** | 3 | 1 | 2 | 6 |
| **Kagawa 2015** | 3 | 2 | 3 | 8 |
| **Kim 2014** | 3 | 2 | 3 | 8 |
| **Kim 2018** | 2 | 1 | 2 | 5 |
| **Lee 2016** | 3 | 2 | 3 | 8 |
| **Maekawa 2013** | 3 | 2 | 3 | 8 |
| **Mecklenburg 2020** | 2 | 2 | 3 | 7 |
| **Otani 2018** | 3 | 2 | 3 | 8 |
| **Pang 2017** | 2 | 4 | 3 | 9 |
| **Ryu 2019** | 3 | 2 | 3 | 8 |
| **Sakamoto 2014** | 3 | 3 | 2 | 8 |
| **Schober 2017** | 3 | 2 | 2 | 7 |
| **Yukawa 2017** | 3 | 2 | 1 | 6 |

**Supplementary** **Table 1.** Quality of each retrospective trial was assessed with the NEWCASTLE-OTTAWA QUALITY ASSESSMENT SCALE CASE CONTROL STUDIES

| Study name | Selection | Comparability | Outcome | NOS score |
| --- | --- | --- | --- | --- |
| **Goto 2018** | 3 | 3 | 2 | 8 |
| **Han 2019** | 4 | 2 | 2 | 8 |
| **Jouffroy 2017** | 3 | 2 | 2 | 7 |
| **Nagao 2010** | 3 | 3 | 2 | 8 |
| **Nagao 2014** | 3 | 3 | 2 | 8 |

**Supplementary** **Table 2.** Quality of each cohort trial was assessed with the NEWCASTLE-OTTAWA ASSESSMENT SCALE COHORT STUDIES


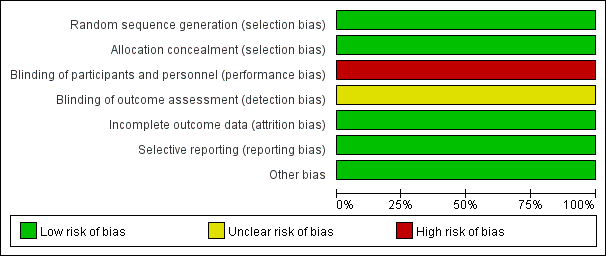


**Supplementary Figure 1.** Risk of bias for Pang 2016


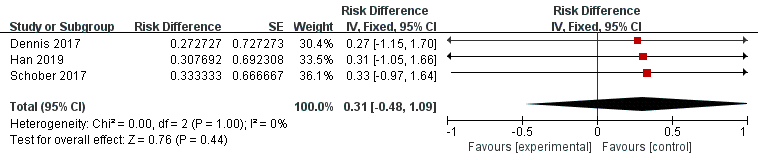


**Supplementary Figure 2.** Single-arm forest plot for survival of temperature>34℃


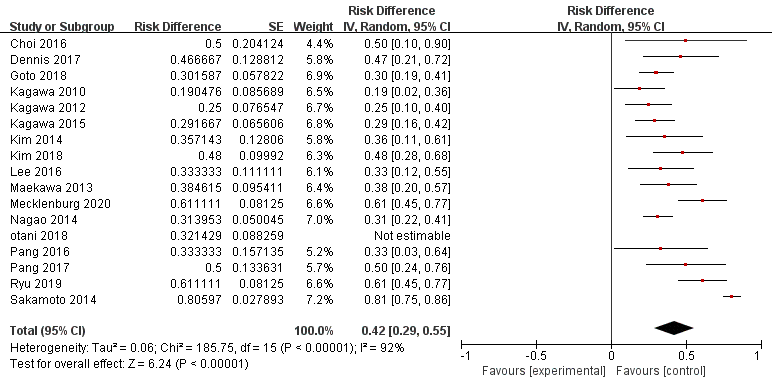


**Supplementary Figure 3.** Single-arm forest plot for survival of temperature≤34℃


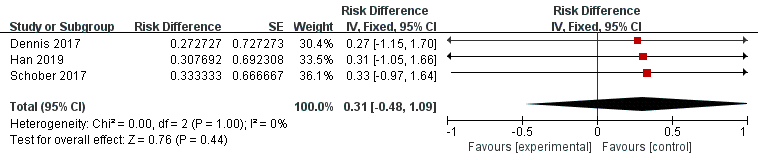
**Supplementary Figure 4.** Single-arm forest plot for favorable neurologic outcomes of temperature>34℃


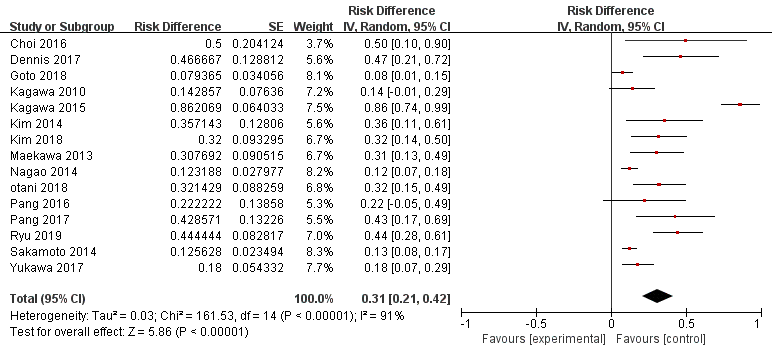


**Supplementary Figure 5.** Single-arm forest plot for favorable neurologic outcomes of temperature≤34℃


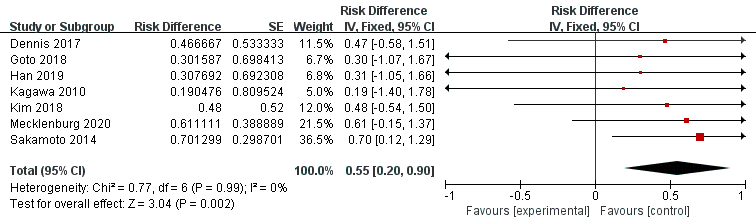


**Supplementary Figure 6.** Single-arm forest plot for survival of combining therapeutic hypothermia before return of spontaneous circulation


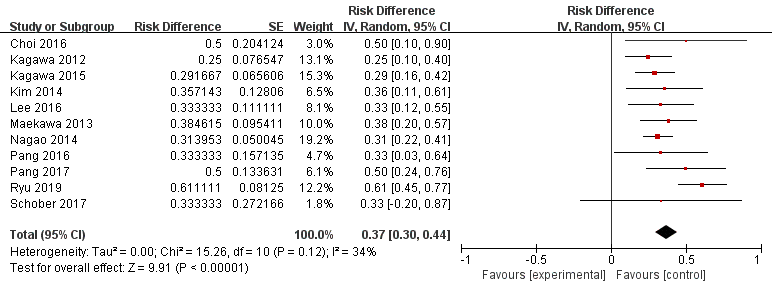
**Supplementary Figure 7.** Single-arm forest plot for survival of combining therapeutic hypothermia after return of spontaneous circulation


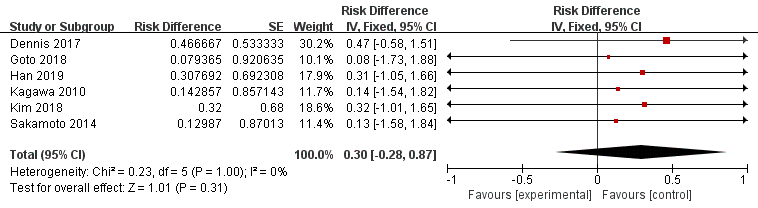


**Supplementary Figure 8.** Single-arm forest plot for favorable neurologic outcomes of combining therapeutic hypothermia before return of spontaneous circulation


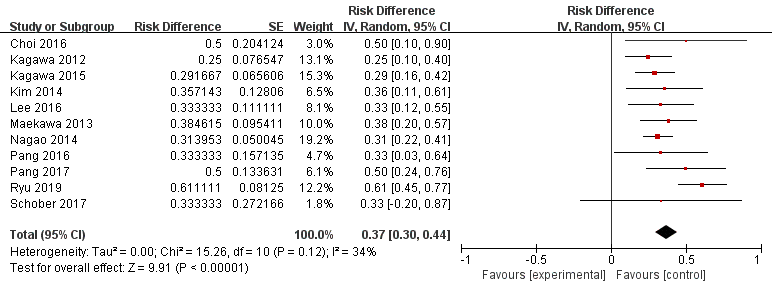


**Supplementary Figure 9.** Single-arm forest plot for favorable neurologic outcomes of combining therapeutic hypothermia after return of spontaneous circulation


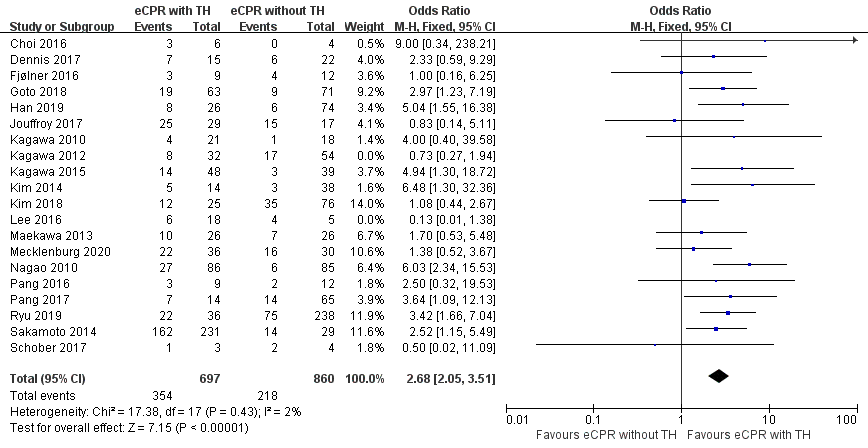


**Supplementary Figure 10.** Forest plot for sensitive analysis

**Search strategy:**

((“Hypothermia, Induced”[Mesh] or Therapeutic Hypothermia or Hypothermia, Therapeutic or Targeted Temperature Management or Targeted Temperature Managements or Induced Hypothermia or Moderate Hypothermia, Induced or Induced Moderate Hypothermia or Induced Moderate Hypothermias or Moderate Hypothermias, Induced or Mild Hypothermia, Induced or Induced Mild Hypothermia or Induced Mild Hypothermias or Mild Hypothermias, Induced) or (“Hypothermia”[Mesh] or Hypothermias or Hypothermia, Accidental or Accidental Hypothermia or Accidental Hypothermias or Hypothermias, Accidental)) and (“Extracorporeal Membrane oxygenation”[Mesh] or Extracorporeal Membrane Oxygenations or Membrane Oxygenation, Extracorporeal or Oxygenation, Extracorporeal Membrane or ECMO Treatment or ECMO Treatments or Treatment, ECMO or ECLS Treatment or ECLS Treatments or Treatment, ECLS or ECMO Extracorporeal Membrane Oxygenation or Extracorporeal Life Support or Extracorporeal Life Supports or Life Support, Extracorporeal or Venoarterial ECMO or ECMO, Venoarterial or Venoarterial ECMOs or Venoarterial Extracorporeal or Membrane Oxygenation) and ((“Cardiopulmonary resuscitation”[Mesh] or Resuscitation, Cardiopulmonary or CPR or Cardio-Pulmonary Resuscitation or Cardio Pulmonary Resuscitation or Resuscitation, Cardio-Pulmonary or Code Blue or Mouth-to-Mouth Resuscitation or Mouth to Mouth Resuscitation or Mouth-to-Mouth Resuscitations or Resuscitation, Mouth-to-Mouth or Resuscitations, Mouth-to-Mouth or Basic Cardiac Life Support or Life Support, Basic Cardiac) or (“Heart Arrest”[Mesh] or Arrest, Heart or Cardiac Arrest or Arrest, Cardiac or Asystole or Asystoles or Cardiopulmonary Arrest or Arrest, Cardiopulmonary))


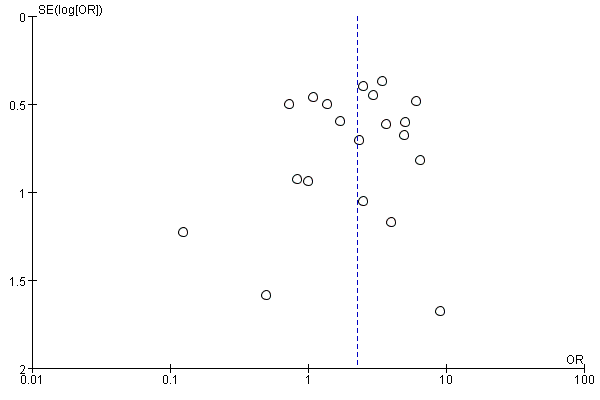


**Supplementary Figure 11.** Funnel plot for survival at discharge or 28 days


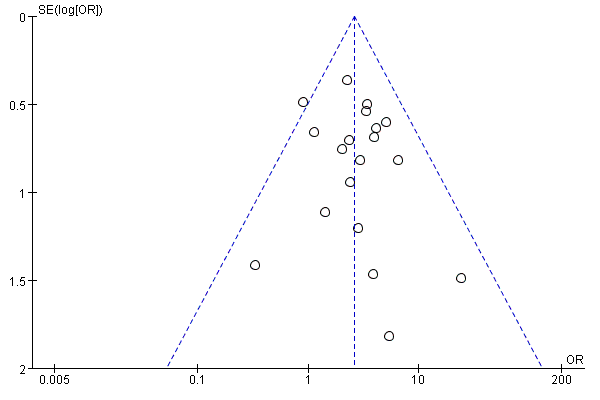


**Supplementary Figure 12.** Funnel plot for favorable neurologic outcomes at discharge or 28 days
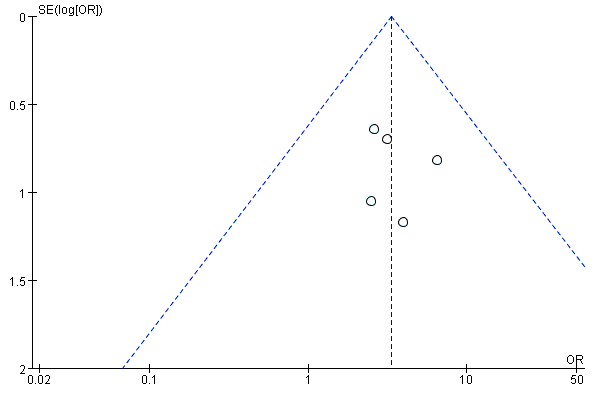


**Supplementary Figure 13.** Funnel plot for survival at 3 months


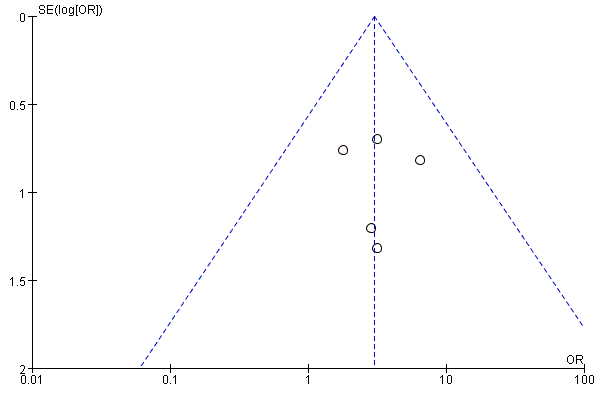


**Supplementary Figure 14.** Funnel plot for favorable neurologic outcomes at 3 months
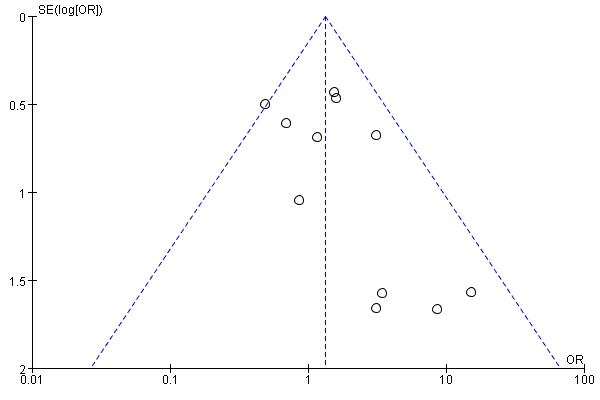


**Supplementary Figure 15.** Funnel plot for any bleeding needed medical intervention during admission
